# IoT Forensics: Current Perspectives and Future Directions

**DOI:** 10.3390/s24165210

**Published:** 2024-08-12

**Authors:** Abdulghani Ali Ahmed, Khalid Farhan, Waheb A. Jabbar, Abdulaleem Al-Othmani, Abdullahi Gara Abdulrahman

**Affiliations:** 1 School of Computer Science and Informatics, De Montfort University, The Gateway, Leicester LE1 9BH, UK; abdulaleem@dmu.ac.uk (A.A.-O.); abdullahi.gara@dmu.ac.uk (A.G.A.); 2 School of Computer Science and Engineering, University of New South Wales, Sydney 2164, Australia; khalidalbakri2018@gmail.com; 3 College of Engineering, Faculty of Computing, Engineering and the Built Environment, Birmingham City University, Birmingham B4 7XG, UK

**Keywords:** forensics, IoT security, IoT privacy, evidence, digital investigations, cybercrime

## Abstract

The Internet of Things forensics is a specialised field within digital forensics that focuses on the identification of security incidents, as well as the collection and analysis of evidence with the aim of preventing future attacks on IoT networks. IoT forensics differs from other digital forensic fields due to the unique characteristics of IoT devices, such as limited processing power and connectivity. Although numerous studies are available on IoT forensics, the field is rapidly evolving, and comprehensive surveys are needed to keep up with new developments, emerging threats, and evolving best practices. In this respect, this paper aims to review the state of the art in IoT forensics and discuss the challenges in current investigation techniques. A qualitative analysis of related reviews in the field of IoT forensics has been conducted, identifying key issues and assessing primary obstacles. Despite the variety of topics and approaches, common issues emerge. The majority of these issues are related to the collection and pre-processing of evidence because of the counter-analysis techniques and challenges associated with gathering data from devices and the cloud. Our analysis extends beyond technological problems; it further identifies the procedural problems with preparedness, reporting, and presentation as well as ethical issues. In particular, it provides insights into emerging threats and challenges in IoT forensics, increases awareness and understanding of the importance of IoT forensics in preventing cybercrimes, and ensures the security and privacy of IoT devices and networks. Our findings make a substantial contribution to the field of IoT forensics, as they not only involve a critical analysis of the challenges presented in existing works but also identify numerous problems. These insights will greatly assist researchers in identifying appropriate directions for their future research.

## 1. Introduction

The Internet of Things (IoT) is a network of devices embedded with electronics, software, sensors, and connections, enabling data sharing and communication. It facilitates automation and cooperation across industries, such as healthcare, agriculture, transportation, and manufacturing [1]. However, security and compatibility issues, including insufficient protection, lack of standardisation, and hacking risks, must be addressed to fully realise the IoT’s potential and ensure privacy and data security [2,3]. Moreover, the expanding use of IoT devices in various aspects of life, including criminal activities, and the need to properly manage digital evidence in judicial procedures increase the demand for IoT forensics. IoT forensics involves the extraction, analysis, and preservation of digital evidence from IoT devices for legal proceedings or incident response [4]. Key areas in this field include extracting evidence from IoT devices, analysing network communications, developing specialised tools and methodologies, addressing challenges posed by the diversity of IoT devices, enhancing the reliability and consistency of forensic evidence, and standardising practices in IoT forensics.

IoT forensics plays a crucial role in various applications, including criminal investigations, incident response, and legal processes. It is essential in criminal investigations for extracting vital digital evidence from IoT devices to solve crimes. In incident response, it helps identify the source and extent of security breaches involving connected devices. Legal processes rely on IoT forensics to gather and preserve data that can be presented in court as evidence. Additionally, it ensures the integrity and authenticity of the information obtained from IoT devices for various legal and investigative purposes. IoT forensics is also a multi-phase process involving several critical steps. These steps include seizing and protecting an IoT device to ensure its integrity, extracting data (both volatile and non-volatile) from the device, analysing data and network communications to uncover important information and event linkages, and presenting the analytical results. Data extraction involves gathering information from various IoT devices and storage media. Researchers are addressing the challenges posed by the diversity of IoT devices, such as differences in operating systems, storage systems, and data formats, and are developing solutions to manage these disparities [5]. Other studies focus on improving the reliability and consistency of forensic evidence obtained from IoT devices and establishing standards for IoT forensics. Network communication analysis involves examining network traffic and communication records to understand the behaviour and interactions of devices [6].

One effective approach in IoT forensics is to examine the version number of the operating system (OS) running on an IoT device to identify any associated exploits. This approach can provide valuable insights into the potential security weaknesses and vulnerabilities of the device, aiding forensic investigators in understanding the device’s threat landscape. By leveraging databases of known vulnerabilities and using specialised tools for OS identification, forensic investigators can gain a clearer picture of the device’s security weaknesses. This approach not only aids in the investigation of security incidents but also contributes to the development of more secure IoT systems. However, some IoT devices contain outdated and poorly written code with potential exploits, and most lack mechanisms for updating their firmware [7]. Existing research on IoT forensics is rare and has challenges due to numerous factors. The complexity of IoT systems and devices and the variety of IoT devices and platforms provide substantial obstacles in data extraction and analysis, which can result in differences in data structure and format. One of the most challenging difficulties is the absence of standardisation and interoperability among IoT devices and systems, which makes developing universal forensic procedures difficult. The dynamic nature of IoT devices can also result in a continually changing environment and data sources, which further complicates data extraction and analysis. Another issue is the lack of consistency in IoT forensics, which makes it difficult to replicate and compare results across multiple devices [7]. The complexities of IoT networks and data storage technologies can make storing and interpreting massive amounts of data difficult, particularly when working with distant or distributed devices. 

Research on IoT forensics should broaden its scope to address the limitations identified in current investigations [8]. First, multidisciplinary research in computer science, digital forensics, and legal/ethical issues is required to understand the intricacies of IoT forensics. Second, feasible and scalable forensic techniques need to be developed for effective investigations of large-scale IoT networks. Third, examining the behaviour of IoT devices and network systems in real-world scenarios is critical for understanding and developing forensic approaches. Fourth, research on evidence preservation and data integrity in IoT devices is crucial for ensuring the validity and trustworthiness of the evidence. Fifth, developing standardised testing techniques to evaluate the usefulness of IoT forensic tools and methodologies can increase their dependability and accuracy. Sixth, working with industry and law enforcement to thoroughly understand the demands and requirements of IoT forensics in real-world scenarios can provide useful ideas for the development process. Lastly, research on the privacy and security implications of IoT data collection and processing is critical for ensuring the ethical and legal use of IoT data in forensics.

Several comprehensive reviews have been conducted to survey the current state of IoT forensics. In this section, selected articles from these reviews are deliberated upon, while a comprehensive analysis of the current relevant works is presented in the IoT forensic review section. A review study [7] examines IoT firmware security, encompassing its scope, vulnerabilities, and detection challenges. It provides a comparative analysis of existing vulnerability assessment tools and concludes with recommendations for IoT device vendors and developers to enhance firmware security. However, this study lacks a framework that audits hardware and network connectivity protocols. A review in [9] presents a comprehensive analysis of IoT’s impact on digital forensics, examining previous research efforts from 2010 to 2018. It introduces a 3D framework that includes temporal, spatial, and technical dimensions, offering principles, guidelines, and exploration of tools to standardize digital investigations in the IoT context. Another review explores IoT concepts, digital forensics, and the current status of IoT forensics, focusing on analysing the challenges of the existing studies [10]. It highlights the ongoing necessity for dedicated efforts to effectively address these challenges. The review in [11] thoroughly reviews IoT forensics, emphasising the system’s security challenges. It discusses the significance of artificial intelligence (AI) in this context, alongside exploring opportunities and key prerequisites for effective IoT forensics. This study also digs into open research directions in IoT forensics. Studiawan et al. [12] proposed a survey of diverse techniques used in forensic investigation, along with an exploration of tools that facilitate event log examination. This survey evaluates publicly accessible datasets utilised in research on operating system log forensics. Additionally, it suggests potential future research in the field of operating system log forensics. Table 1 summarises the main contributions and weaknesses of the aforementioned reviews.

In response to the identified weaknesses outlined in the existing literature, this paper makes several contributions aimed at providing a comprehensive and insightful overview of IoT forensics, highlighting its importance and the challenges that researchers and practitioners face in this area. Initially, it outlines a comprehensive overview of IoT forensic techniques, categorising them into several categories including artificial intelligence in IoT forensics, IoT applications, IoT network architecture, cutting-edge digital forensics, and blockchain-based digital forensics. Secondly, this paper also discusses other techniques that have been proposed to investigate forensics in the IoT environment. Thirdly, this study critically analyses the strengths and weaknesses to evaluate the efficiency of the reviewed techniques in terms of artefact extraction, analysis, and reporting. Lastly, the study provides important directions for future research that can help improve the efficiency of existing techniques and enhance their analytical capabilities. The remainder of this paper is structured as follows: Section 2 presents a comprehensive review of IoT forensics. Section 3 describes the IoT forensic layers. Section 4 discusses previous studies on IoT forensics techniques utilising various technologies, including artificial intelligence, advanced digital forensics, and blockchain. Finally, Section 5 outlines the conclusions and suggests future work.

## 2. The Internet of Things (IoT) Forensics

IoT forensics involves the combination of techniques, tools, and resources from all aspects of digital forensic (DF) science to deal with IoT-related crimes. This involves the investigation of embedded devices, connected sensors, cloud services, and applications connected to the embedded devices [13]. The heterogeneous characteristics of this domain are well documented as presenting significant challenges in cyber security research. However, in conventional DF, this heterogeneity complicates the problem. 

A common occurrence in DF investigation is multiple iterations of some or all processes in the investigation, especially with the identification of new sources of evidence during examination and analysis [14]. In the IoT context, multiple iterations would increase the amount of data to be analysed significantly, which is unavoidable. Other views presented by [15] support concurrent processes that could enhance efficiency, effectiveness, and evidence admissibility, thereby reducing the time it takes to analyse evidence. However, on closer critical examination, some drawbacks can be seen. Combining these two techniques (concurrent processing and multiple iteration techniques) could yield positive outcomes. Performance may increase efficiency in small-scale investigations involving independent IoT devices discovered at crime scenes. However, when it comes to conducting large-scale IoT investigations that involve hundreds or thousands of devices, employing this combined technique may prove to be resource-intensive and burdensome. With the IoT devices numbered in the billions, it is unknown how many devices may be implicated in an investigation.

Additionally, resource constraints of smart devices and the competitive business landscape result in numerous challenges to designing and incorporating sophisticated security features [16], which make the IoT infrastructure an attractive target of cyberattacks. This is evident in the proliferation of attack attempts, which can be attributed to the increasing number of IoT devices serving as entry points to networks [17]. Consequently, IoT devices and networks can act as both victims [18] and attackers [13,19]. Furthermore, the cyber physical systems paradigm means that the IoT engages with both virtual and physical worlds concurrently [20], enabling cybercrime to effectively cross from the virtual to the physical environment [21]. It comes as no surprise that a significant amount of cybersecurity research has been devoted to finding solutions by applying technologies such as blockchain [22,23], honeypots [24,25], and machine learning techniques [6,26,27] to mitigate concerns and prevent future occurrences.

In contrast to this, DF science focuses on identifying and reporting deficits in security, investigation procedure, retrieval of artefacts of evidentiary value, reconstruction of events, and discovery of attack vectors during the incident response stages [28]. IoT forensics provides opportunities to improve the integrity and validity of forensic investigations [29] and curb the growth of adversarial threats by enabling timely prosecution [13]. However, DF in the context of IoT investigations presents different challenges that are not encountered in non-IoT investigations. Typically, non-IoT investigations begin by following standard DF processes, starting with the identification of devices linked to a crime. In IoT investigations, however, things become more complicated, especially when physically accessing the relevant “things” is difficult or impossible. Moreover, errors in IoT investigations could result in the inclusion of “things” in the investigation that have no connection to the crime [30].

Finally, as DF science is often dependent on post-incident activities, the most widely adopted approach in research post incident is the development of conceptual frameworks or process models for triage in IoT investigation.

## 3. IoT Forensic Layers

**Figure 1:**
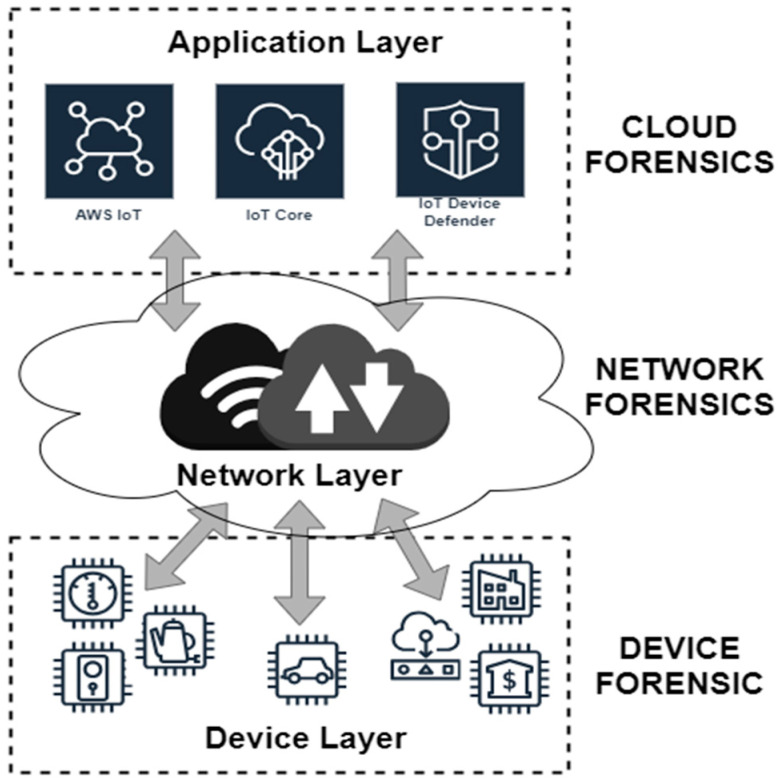
Conceptual model categories for IoT forensics.

IoT forensic investigation often involves considerations of evidentiary data from heterogeneous network domains, which need to be collected together during the investigation process. For this purpose, the research [9] provides three broad layers of the IoT: (i) the IoT devices or sensors, (ii) the internal network, and (iii) the cloud [6,9,31]. A study [32] presented IoT forensics as a “combination of three digital forensic layers”: device, network, and cloud forensics. The aim of their categorisation is to split research into specific sub-problems in the IoT forensics domain. This is illustrated in Figure 1, which presents the categories of IoT directly mapped to the three digital forensic layers. However, IoT systems exist that do not require the cloud, or complex network connections. Edge computing uses a distributed architecture for data processing that is closer to the source of its generation. Though the three forensic layers model does not claim to represent all types of IoT connectivity, it does cater to three common IoT network computing architecture types: the cloud layer, the fog layer, and the edge layer. 

There are IoT devices that, after capturing data, do not send it to the cloud for processing but do some or all of the processing [33,34,35]. Taking into consideration the distributed data processing in edge and fog computing, the IoT forensic categorisation by [32] three forensic layers may not be suitable in practice, but is useful as a conceptual model for all IoT forensic categories. In spite of its limitations, this categorisation enables outlining requirements and procedures for forensic investigations for how, what, where, when, and why evidence should be collected at different levels of the IoT infrastructure.

- Device Layer Forensics: IoT devices are versatile, and there are no universal forensic methods. Evidence may be acquired from the local memory of IoT devices, such as audio, images, videos, and log files. This data, which includes user behaviour, sensor data, heart rate data, configuration data, telemetry data, and device states, comes from devices such as CCTV cameras, medical implants, smart home appliances, networked vehicles, and UAVs.
- Network Layer Forensics: The network layer of IoT comprises various networks connecting devices to each other and the internet, such as PANs, BANs, WANs, HANs, and LANs. Leveraging the logging and auditing capabilities of these networks can collect legally admissible evidence to trace users within the IoT ecosystem [10].
- Cloud Layer Forensics: Due to the storage and computational constraints of IoT devices, cloud computing offers advantages such as on-demand accessibility and processing capacity. Data generated by IoT devices are transmitted to the cloud for storage and processing, making the cloud crucial in IoT forensics. Client-centric artefacts and other relevant data, such as authentication, access, system, database, and application logs, can be extracted from the cloud to reconstruct cases [31].

## 4. IoT Forensic Review

**Figure 2:**
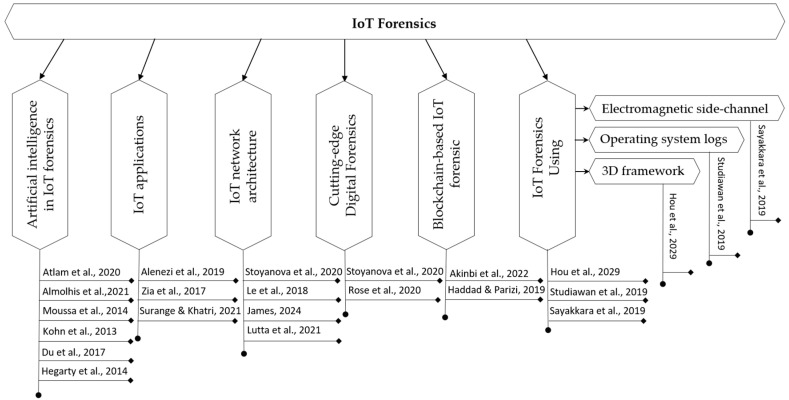
An illustration of the various categories into which current IoT forensics techniques have been classified [6,9,11,12,31,36,37,38,39,40,41,42,43,44,45,46,47,48,49].

This section comprehensively reviews the present techniques and explores future areas of research relevant to IoT forensics. The existing IoT forensics techniques have been classified into several categories, as illustrated in Figure 2. A comprehensive review is provided in the following subsections.

### 4.1. Artificial Intelligence in IoT Forensics

An examination of artificial intelligence in IoT forensics commenced with an extensive exploration of IoT security, emphasising the crucial role of artificial intelligence (AI) in this field [11]. The review covered current research, recent studies, emerging possibilities, and essential requirements for effective IoT forensics. The issues and potential solutions of IoT forensics were also emphasised. Afterward, the issue of open IoT forensic research paths was presented. Digital forensics was explained by describing digital forensic inquiry. The differences among traditional, cloud, and IoT forensics and the use of AI in IoT forensics were also highlighted to provide a comprehensive overview of IoT forensics. The most recent and cutting-edge IoT forensics investigations, tools, and applications were then presented. The discussion of prospective future study fields for IoT forensics included obstacles, possible solutions, and prospects. Several lingering difficulties in IoT forensics that require additional research to increase the pace at which IoT devices are employed were pinpointed. Some of these challenges were detailed in the study to understand the concerns and provide appropriate solutions to them. This study concluded that further research is needed to establish frameworks for IoT forensic investigations that can successfully handle the vast volumes of data produced by heterogeneous IoT devices. Furthermore, IoT manufacturers must consider the forensic preparedness of their IoT devices/products during the design phase. In the future, they need to develop a forensic investigation framework for IoT devices in smart homes to reveal existing smart home device evidence that can be effectively used in digital investigations. 

Another study relevant to AI forensics analysed the most notable technical papers on IoT forensics by 2021 and highlighted some of the IoT forensic issues [36]. It provided a list of needs that an IoT forensic process model should address based on these problems. Furthermore, the IoT forensic process models described in the literature were analysed based on the inferred needs to identify the remaining gaps. The capacity of these IoT devices to collect data from many types of sensors is an important feature. Much of the information gathered may be deemed personal by the owners of the devices. IoT architectures include tracking and identification technologies integrated through actuator and sensor networks to allow communication between distributed intelligent objects, such as wearable smart devices, that may collect location information without the user’s consent [37,38].

The best practices and guiding principles of digital forensics and any sub-discipline, such as IoT forensics, require that the tools used for the investigation and the process of conducting the investigation be reviewed, calibrated, verified, and approved so that they can be replicated independently. This task helps ensure that digital evidence gathered in a forensically sound manner can be accepted in court. As a result, process models are critical to speed up the investigation and address the challenges investigators are experiencing, particularly with new technologies, to standardise and capture the process of conducting digital investigations [36,39,40]. Different tools required for investigating various technologies have been developed based on commonly recognised concepts.

### 4.2. IoT Applications

The paper [41] focuses on developing effective digital forensics techniques designed to trace the origins of attacks during security breaches and ensure that perpetrators are held accountable with reliable digital evidence. A significant challenge in this area is the diverse nature of devices within IoT systems and the absence of unified standards. The authors explore digital forensics from an IoT perspective, emphasising the necessity of application-specific forensics alongside traditional methods. They examine the top three IoT applications and present a model that integrates both conventional and application-specific forensic processes. The proposed model aims to enhance the collection, examination, analysis, and reporting of robust forensic evidence in IoT-related investigations.

The review paper [42] summarised recent advances in IoT forensics and attempted to identify gaps, problems, and the field’s scope. It is noted that current digital forensic methodologies are inadequate for IoT systems due to socio-technical challenges. Despite numerous IoT forensic frameworks, none address the full range of data, applications, or jurisdictions that might be involved in forensic inquiries. The authors emphasised the need for a comprehensive framework that considers these factors. They acknowledged the difficulty of incorporating various jurisdictional requirements but suggested considering commonalities across frameworks to simplify and improve the process.

The IoT forensics and state-of-the-art approaches to IoT forensics were reviewed in the paper [10]. This paper evaluated the current literature of IoT forensics and the potential solutions offered in recent works. They analysed the issues that IoT forensics is facing, as documented in recent literature. IoT forensics-related challenges were also discussed, followed by an overview of prospective future research fields. Further research on how to achieve IoT forensic preparation needs to be conducted in the future to enable organisations to undertake digital investigations.

### 4.3. IoT Network Architecture

While reviewing another study [43], they discovered that the paper offered an architecture that splits the IoT network into three zones: internal, middleware, and external. By combining the zones, the researchers applied the triaging concept to their model. According to the researchers, their methodology is suitable for internal incident responders. The model excludes IoT devices and applications from the scope of IoT forensic investigation. Instead, it operates at the network layer of the IoT ecosystem. Furthermore, the model does not include user privacy problems and, as a result, does not provide ways to secure user identification in real data collected for analysis.

A blockchain-based IoT forensic approach that protects identity privacy throughout the evidence’s lifespan is presented in [44]. In this approach, the collection phases are determined by whether enough evidence has been collected and whether the victim has sufficient evidence. One flaw of this strategy is that it focuses on evidence collection after other critical parts of the IoT investigation have been completed. A consumer must be notified about the technique, policies, and practices of forensic analysis to which his or her data will be submitted under the openness, transparency, and notice requirements. Similarly, the customer must have access to his or her data throughout the investigation process. The accountability requirement encourages investigators to adhere to privacy regulations established for gathering and analysing evidence. The information security control requirement maintains the protection of collected personal data against unauthorised access, loss, or alteration. The compliance criterion includes the development of an auditing tool to ensure that the entire investigation process adheres to privacy standards [43].

Another study [6] reviewed the state of digital forensic process models specific to the IoT context. It defined the requirements that an IoT forensic process model should fulfil to be applicable to IoT organisations. These requirements were gathered from the literature to address IoT forensic concerns and challenges encountered by digital forensic investigators. This study also evaluated current cloud forensic process models in the literature against the identified requirements and discussed the gaps in standardising cloud forensics.

A systematic literature review (SLR) studied the latest advancements in IoT forensic research [45]. This SLR focused on the fundamentals of IoT, IoT applications, the primary impacts on IoT forensics, and the applicability of various methodologies applicable to IoT forensics. The SLR identified research challenges and concluded that the majority of existing studies are theoretical rather than applied. To address the identified challenges, realistic solutions are necessary. Table 2 provides a concise overview of the key findings and drawbacks outlined in the review conducted by [10,11,41,42,45] while also highlighting the outstanding difficulties and requirements for IoT forensics.

### 4.4. Cutting-Edge IoT Forensics

The advancements of new technologies, such as low-cost image/video recording and information processing methods (e.g., artificial intelligence and machine learning), have posed increased challenges for forensic investigators. Thus, the primary objective of the study conducted in [6] was to review cutting-edge digital forensic methodologies and examine security vulnerabilities in IoT devices from a forensic perspective. In particular, this study presented a brief review of the fundamental challenges, philosophical foundations, and emerging research areas in IoT forensics. It emphasised the importance of standardising forensic techniques as a crucial step for generating jurisdictional forensic reports and establishing best practices for cybersecurity. 

Public organisations and legal authorities should be aware that IoT forensics is still trailing behind other well-established areas of digital forensics, and it demands further funding and research. This paper also concludes the need to expand and adapt traditional forensic approaches in order to preserve legally admissible evidence by examining current issues and challenges in IoT forensics. In addition, explicit IoT security principles and widely recognised standards are necessary. 

The research conducted in [46] explored cutting-edge digital forensic techniques for audiovisual biometric data, which can be applied in smart city applications. Smart technology, whether in the form of a smart economy or smart utilities, has become an integral component of urban civilisation, offering intelligent, practical, and secure solutions to a wide range of daily services. Smart cities are composed of a network of interconnected IoT devices that must communicate with each other and with humans. Biometric authentication, where the device validates the intended user by using biometric data collected from the user, can be utilised to protect human–machine interaction. However, it is important to ensure security and privacy when dealing with biometric data. Additionally, this study examined existing digital image, audio, and video-based forensic approaches applied to biometric data. It discussed the current challenges in forensic systems, with particular focus on the challenges posed by deepfake audios and videos.

### 4.5. Blockchain-Based IoT Forensics

Multiple blockchain-based models have recently been established for forensic investigations in the IoT. According to the research conducted in [47], these models employ the inherent capabilities of blockchain, ensuring the chain of custody, privacy, integrity, provenance, traceability, and verification of the evidence maintained during the investigation process. The majority of the models are based on permissioned blockchains. However, the same architectures could be applied in permissionless blockchains, such as Bitcoin, Ethereum, Algorand, Avalanche, and Polkadot. The research conducted also evaluated the effectiveness of various proposed models and proof-of-concept prototypes based on their outcomes and performance metrics. The conducted evaluation led to the identification of concerns, unsolved issues, and potential topics for future research. 

Due to the lack of prototypes established to indicate their practical applications, there is a noticeable absence of descriptive studies on these blockchain-based IoT forensic models. Future research is recommended to conduct an empirical examination of the security aspects of current blockchain-based IoT forensic investigation models, as well as other emerging models. Another survey [48] reviews IoT security concerns, restrictions, demands, and existing and potential solutions. In the survey’s taxonomy, a three-layer IoT architecture is utilised as a frame of reference to define the security requirements and qualities for each layer. The key value of this survey is its assessment of potential IoT security vulnerabilities and concerns from an architectural standpoint. The three-layer architecture is then used to assist readers in understanding how to adopt best practices to mitigate existing IoT security concerns. Table 3 provides a summary of the key findings and insights for future research derived from the review conducted by [6,47,48]. 

### 4.6. Other IoT Forensics

*A.*  *IoT forensics using Electromagnetic side-channel*

The authors of our third reviewed paper [49] provided a survey of the literature on electromagnetic (EM) side-channel analysis to enhance digital forensic investigations of IoT devices. In EM side-channel analysis, which is a way of listening in on computer activity and data handling, unintentional EM emissions are used. Given that EM side-channel assaults do not need and must not result in any changes to the target device, they are an appropriate technique for digital forensic investigations. Studies on various EM side-channel analysis attack methodologies are examined and selected based on their potential for usage in scenarios involving the evaluation of IoT devices. The background research information is used to identify potential future uses of the technology in the digital forensic analysis of IoT devices, which may produce a wide spectrum of currently halted digital investigations.

Traditional digital forensics examines the file storage, log files, network traces, and so on that suspects leave behind on digital devices. Live data forensics can be used in systems that require complicated investigation methods and skills. The regular job of digital forensic investigators must evolve as computer systems shift from soft platforms that are less concerned with privacy and security to hardened platforms that are designed with security in mind from the start. Cryptographically protected storage systems are one of the most notable barriers to successful digital forensic investigation. From the aspect of security, EM side-channel analysis has been demonstrated to be a potential door opener for cryptographically secured data storage and communication, and it may be developed upon and utilised in digital forensic applications.

The goal of [49] was to modify the technique to help with digital forensic investigations on IoT devices. To achieve this goal, a comprehensive review of the literature on EM side-channel attacks was performed. Although numerous mitigation measures have been established and implemented to guard against EM side-channel attacks, current research demonstrates that these efforts have not been successful in diminishing the prevalence of this attack vector. EM side-channel analysis is still in its infancy in digital forensic applications. When used not just to extract security keys but also to identify inadvertent data loss, it needs court-admissible, forensically sound processing. However, this technology has the potential to greatly affect the industry and expedite the development of previously stalled investigations involving IoT devices and generally secure computer systems.

*B.*  *IoT forensic using 3D framework*

The authors of [9] combined previous academics’ (2010–2018) research efforts and examined the implications of the IoT in digital forensics. They examined the IoT forensic environment and performed a 3D study of the area. A 3D framework involving temporal, geographical, and technical dimensions was created. The geographical component analysis shows how to discover evidence sources in an IoT scenario, and the time dimension discusses the standard digital forensic procedure. The two aspects work to establish standards and recommendations for standardising digital investigations in the IoT. The technological aspect guides research tools and procedures to ensure the application of digital forensics in the ever-changing IoT context. 

The authors in [9] identified unsolved issues and provided positive recommendations to encourage further study. They reported that key IoT characteristics have a considerable influence on traditional digital forensics. Through ubiquitous sensing, the number, diversity, and sources of prospective evidence are enhanced. When dynamic changes occur, determining the persons involved and defining the case’s limits become increasingly difficult. The challenge of distinguishing who is responsible emerges with automated execution. Finding and collecting volatile or non-volatile data from an IoT context is difficult due to the environment’s restricted resources. Diversification considerably increases the burden of researchers. Given the unique security aspects of the IoT, erasing and changing potential evidence is difficult. The number of studies on the subject increases as public awareness of IoT forensics intensifies. To obtain a clear perspective of the research directions and state of research in this domain, the authors defined the landscape of IoT forensics within a 3D framework consisting of geographical, temporal, and technical dimensions. A smart house was adopted as an example to demonstrate 3D IoT forensics. Existing research projects were meticulously assessed within the 3D framework with the intention of providing recommendations for forensic researchers and practitioners.

To guide forensic investigations in the IoT paradigm, forensic models should follow the core forensic approach, and they should be changed to consider the IoT environment in practical use. From a geographical perspective, investigators must have access to a plethora of potential evidence sources in the IoT, such as devices, networks, and clouds. Re-constructing an event scene necessitates the integration of evidence from many data sources. To deal with new data sources, new forensic tools/techniques and forensic readiness systems for IoT contexts should incorporate real-time logging, volatile data processing, and support for a range of hardware and file systems.

*C.*  *IoT forensics using operating system logs*

A thorough analysis of the literature on forensic inspection of operating system logs was conducted in [12]. The authors provided a classification of the many approaches used in this subject and discussed the technologies that aid in event log analysis. This study also examined the publicly available datasets used in operating system log forensic research. They concluded their review by suggesting future work directions for operating system log forensics. They also provided a detailed examination of this corpus of evidence. This article examines several forensic event log analysis approaches, with a focus on operating system (OS) logs. This study constructed a taxonomy based on a generic investigation method that included event log recovery, event correlation, event reconstruction, and visualisation. They categorised current articles by using a generic forensic framework. The advantages and disadvantages of the techniques in each area were discussed in depth. The authors also presented an in-depth review of OS log forensic research. This work is organised in accordance with a framework for digital forensic investigation.

The researchers in this paper also explained the tactics utilised in the literature for each phase and discussed their pros and cons, including the tools needed to study OS logs. Detailed explanations of publicly available datasets were also provided. One of the primary challenges identified was encouraging the research community to utilise shared datasets to enable the evaluation and comparison of proposed solutions for efficiency. The study comprehensively addresses all legal, privacy, and cloud security issues, along with other critical complexities encountered in intricate investigations involving the IoT. Additionally, the study provides an overview of both past and current theoretical frameworks in digital forensics, with a particular focus on frameworks leveraging decentralised blockchain-based technology to safeguard evidence integrity. Furthermore, the study explores several innovative data reduction and forensic intelligence approaches, including the emerging forensics-as-a-service (FaaS) model. Finally, the analysis of current research trends and unresolved issues underscores the importance of proactive forensic readiness initiatives and widely recognised standards.

The use of open-source technology is also critical in the development of cutting-edge approaches for OS log study. Throughout history, attackers have used increasingly intricate and advanced methods to circumvent forensic techniques and instruments. Thus, sophisticated approaches are necessary to detect and analyse computer system risks. A succinct overview of the primary findings and challenges analysed in the review conducted by [9,12,49] as well as outstanding issues in OS log forensic research are outlined in Table 4.

## 5. Conclusions and Future Work

An efficient and adequate digital forensic investigation in the IoT networks is required to protect IoT applications from cybercrimes and trace their sources. This requires the development of frameworks particularly designed to tackle the challenges posed by IoT devices. These frameworks should establish standardised procedures, methodologies, and approaches for investigating digital evidence in cybercrimes related to IoT. By adapting forensics standards, incorporating machine learning techniques, and addressing privacy concerns, these frameworks may contribute to the advancement of IoT forensic investigations. This study conducted a comprehensive analysis of IoT forensics, with a specific focus on current challenges and areas for future research. It dived into the challenges that investigators face due to the heterogeneous nature of IoT networks and the proliferation of vulnerable devices. As part of this study, IoT forensics layers are presented, describing the collection of specific types of evidence from IoT devices, networks, and cloud storage to reconstruct cases and trace users within the ecosystem.

Through a critical analysis, this study explores the challenges and complexities hindering forensic investigators from conducting authentic investigations in IoT networks. It highlights various unresolved challenges in IoT forensics, which emphasises the need for further investigation in order to stimulate widespread adoption of IoT devices. Additionally, it examines potential solutions and future prospects of IoT forensics while outlining research directions for the future. Future work entails the development of a robust forensic investigation framework tailored for IoT devices, leveraging advanced technology to establish a reliable method of extracting evidence from modern smart home devices. Further research in IoT forensics will also look into new forensic tools and techniques, identify data sources, and seek to integrate IoT forensics with broader areas of cybersecurity and digital forensics.

## Figures and Tables

**Table 1:** Related works’ contributions and weaknesses.

Study	Contributions	Weaknesses
[7]	Highlights the significance of IoT firmware security and offers an updated assessment of vulnerabilities and solutions in this domain.	Insufficient auditing of hardware and network connectivity protocols. This study does not focus on issues relevant to IoT forensics.
[9]	The study offers a holistic overview of IoT digital forensics, identifying open issues and proposing suggestions for future research.	A thorough critical analysis has not adequately been carried out to pinpoint the strengths and weaknesses of the reviewed studies. Furthermore, the identified weaknesses have not been extensively discussed to determine their potential as topics for future research.
[10]	The analysis explores digital forensics and current issues in the IoT forensics. It highlights the researcher’s efforts to effectively address these issues.	The state-of-the-art section is concise and lacks a comprehensive description or categorisation of existing works. Additionally, the weaknesses of the reviewed studies have not undergone critical analysis.
[11]	This review provides a comprehensive examination of IoT forensics, emphasising the significance of artificial intelligence (AI). Additionally, it outlines future research directions in the field.	A general discussion has been held regarding the requirements for successful IoT forensics and the challenges and suggested solutions within the field. However, the specific weaknesses of each study covered in this paper’s review process have not undergone in-depth critical analysis.
[12]	An analysis of forensic investigation techniques and tools applied to operating system aiding event log analysis. It also includes an assessment of available datasets and recommendations for future research.	The proposed approaches and tools for forensics are not primarily designed for IoT environments. As a result, there is a potential need to adjust them to suit the specific requirements of IoT forensics, particularly in the context of event log analysis.

**Table 2:** Findings and directions for future research in IoT forensics focusing on AI, applications, and network architecture.

Ref.	Research Findings	Directions for Future Research
[10]	A comprehensive overview of IoT forensics and challenges in current literature. The general goal of this study is to assess both the IoT and digital forensic sectors, pinpoint associated issues, and propose directions for future research endeavours.	This work identifies potential areas relevant to IoT forensics, such as IoT forensic procedures, multi-jurisdictions, big IoT data analysis, anti-forensic data pooling, and IoT forensic readiness, as future research directions.
[11]	An overview of IoT forensics underscores the necessity of AI integration for successful IoT forensics. An emphasis on critical factors for conducting thorough forensic investigations.	Future research can fucus on creating a forensic investigation framework for identifying evidence from current smart home equipment. Additionally, it can explore potential challenges and solutions associated with the integration of AI into IoT forensics.
[41]	An exploration of the necessity for application-specific forensics alongside traditional methods. An examination of the top three IoT applications and the presentation of a model that integrates both conventional and application-specific forensic processes.	Future research can be conducted to study the diverse nature of devices within IoT systems and the absence of unified standards.
[42]	A review of recent advancements to identify gaps and difficulties of the field’s research. Findings indicating that current digital forensic approaches are inappropriate for forensic analysis in IoT systems due to socio-technical difficulties.	Exploring challenges accompanying IoT Integration into society. Thus, issues related to IoT privacy issues, multiple jurisdictions, forensic analysis with big data techniques, and dealing with anti-forensic techniques can be important directions for future research.
[45]	This article covers three key areas: data recovery and acquisition, file systems, and data analysis. It discusses the techniques used to capture digital evidence from the storage media, file systems, and memory of mobile devices.	Further research is required to develop intelligent and efficient tools that are scientifically validated to guide digital investigations in complex IoT environments.

**Table 3:** Findings and directions for future research in IoT forensics focusing on cutting-edge digital forensics and blockchain-based IoT forensics.

Ref.	Research Findings	Directions for Future Research
[6]	This study summarises previous and present theoretical frameworks that have been proposed to maintain the integrity of digital evidence using decentralised blockchain-based technologies. The study also discusses various interesting cross-cutting data reduction and forensic intelligence methodologies, as well as the current forensics-as-a-service (FaaS) model.	Future research can be conducted to study recent challenges arising in forthcoming forensic investigations that rely extensively on video evidence. Advance methodologies to address privacy concerns and integrate cross-disciplinary computational techniques, including AI predictive analytics, run-time verification, and adaptive data collection.
[47]	IoT investigation frameworks and models integrating blockchain technology, aimed at ensuring the chain of custody for forensic evidence while upholding privacy, integrity, and preservation. Through an SLR encompassing primary papers up to late 2021, this research contributes to the existing body of knowledge.	Further research is required to ensure the establishment of a reliable blockchain-based IoT forensic investigation procedure, capable of thoroughly addressing potential challenges and obstacles. Future research could also incorporate an empirical assessment of the security measures implemented in blockchain-based IoT forensic investigation models, as well as other recent models.
[48]	This survey presents an architectural classification of IoT security threats and issues, providing insights to comprehend and implement best practices for addressing security risks. Additionally, it evaluates security issues and proposes solutions within IoT contexts, presenting a taxonomy for security challenges based on the three-layer architecture.	This survey primarily reviews research conducted before 2019. However, given the growth of technology and the escalating threats, it is imperative to continue conducting this type of research to ensure that it remains current and up to date.

**Table 4:** Findings and ideas for future research in IoT forensics focusing on electromagnetic side-channel, 3D framework environment, and operating system logs.

Ref.	Research Findings	Directions for Future Research
[9]	A summary of IoT forensic research conducted from 2010 to 2018 and a brief history of the field’s development. A 3D framework-based sketch of the IoT forensic ecosystem. Outlining unresolved issues in the IoT forensic sector and offering relevant recommendations.	Future research can focus on identifying fundamental rules and directions through the execution of common forensic procedures in IoT forensics.
[12]	The articles in this research included a wide range of subjects, such as event log security and recovery, event reconstruction and correlation, event anomalies, and visualisation. The authors provided a list of approaches that are already in use, a critical overview, and an analysis of each method’s benefits and drawbacks. Given that OS logs are frequently found when evidence is retrieved from a forensic disc image, this study explored techniques for conducting forensic analysis of OS logs. The article also discussed OS log-focused public datasets and forensic tools.	Future research may enhance the security of event logs by combining encryption, centralisation, and hardware-supported designs. It is also stated that event log forensics may be a direction for future studies.
[49]	A thorough examination of EM side-channel attacks as a method to support digital forensic investigations on IoT devices.	EM side-channel methods are rarely utilised for digital forensics; therefore, more research to identify their tools and standards would be beneficial.

## Data Availability

The data we used is public and available online.
